# Transcriptome profiling of immune rejection mechanisms in a porcine vascularized composite allotransplantation model

**DOI:** 10.3389/fimmu.2024.1390163

**Published:** 2024-05-21

**Authors:** Lei Zhang, Isabel Arenas Hoyos, Anja Helmer, Yara Banz, Cédric Zubler, Ioana Lese, Stefanie Hirsiger, Mihai Constantinescu, Robert Rieben, Mitra Gultom, Radu Olariu

**Affiliations:** ^1^ Department of Plastic and Hand Surgery, Inselspital University Hospital Bern, Bern, Switzerland; ^2^ Department for BioMedical Research, Faculty of Medicine, University of Bern, Bern, Switzerland; ^3^ Institute of Pathology, Faculty of Medicine, University of Bern, Bern, Switzerland

**Keywords:** vascularized composite allotransplantation (VCA), immune rejection, transcriptome profiling, RNA sequencing, bioinformatics analysis, large animal model

## Abstract

**Background:**

Vascularized composite allotransplantation (VCA) offers the potential for a biological, functional reconstruction in individuals with limb loss or facial disfigurement. Yet, it faces substantial challenges due to heightened immune rejection rates compared to solid organ transplants. A deep understanding of the genetic and immunological drivers of VCA rejection is essential to improve VCA outcomes

**Methods:**

Heterotopic porcine hindlimb VCA models were established and followed until reaching the endpoint. Skin and muscle samples were obtained from VCA transplant recipient pigs for histological assessments and RNA sequencing analysis. The rejection groups included recipients with moderate pathological rejection, treated locally with tacrolimus encapsulated in triglycerol-monostearate gel (TGMS-TAC), as well as recipients with severe end-stage rejection presenting evident necrosis. Healthy donor tissue served as controls. Bioinformatics analysis, immunofluorescence, and electron microscopy were utilized to examine gene expression patterns and the expression of immune response markers.

**Results:**

Our comprehensive analyses encompassed differentially expressed genes, Gene Ontology, and Kyoto Encyclopedia of Genes and Genomes pathways, spanning various composite tissues including skin and muscle, in comparison to the healthy control group. The analysis revealed a consistency and reproducibility in alignment with the pathological rejection grading. Genes and pathways associated with innate immunity, notably pattern recognition receptors (PRRs), damage-associated molecular patterns (DAMPs), and antigen processing and presentation pathways, exhibited upregulation in the VCA rejection groups compared to the healthy controls. Our investigation identified significant shifts in gene expression related to cytokines, chemokines, complement pathways, and diverse immune cell types, with CD8 T cells and macrophages notably enriched in the VCA rejection tissues. Mechanisms of cell death, such as apoptosis, necroptosis and ferroptosis were observed and coexisted in rejected tissues.

**Conclusion:**

Our study provides insights into the genetic profile of tissue rejection in the porcine VCA model. We comprehensively analyze the molecular landscape of immune rejection mechanisms, from innate immunity activation to critical stages such as antigen recognition, cytotoxic rejection, and cell death. This research advances our understanding of graft rejection mechanisms and offers potential for improving diagnostic and therapeutic strategies to enhance the long-term success of VCA.

## Introduction

Vascularized composite allotransplantation (VCA) represents a beacon of hope for individuals who have endured limb loss or severe disfigurement when traditional reconstructive techniques fall short (1–3). VCA involves the transplantation of composite tissues such as skin, muscle, bone, blood vessels, and nerves (4). The potential applications of VCA, such as face and limb transplants, are aimed at reconstructing defects resulting from trauma, tumor resection or total limb loss (5, 6). To date, more than one hundred upper extremity and 48 face transplantations have been performed worldwide (6, 7). While VCA holds immense potential for transforming lives, the issue of immune rejection presents a formidable obstacle, hindering widespread adoption of this technique. In comparison to solid organ transplantation, immune rejection in VCA poses distinct challenges (5, 6). The International Registry on Hand and Composite Tissue Allotransplantation (IRHCTT) reports that more than 80% of upper extremity transplant recipients experience at least one episode of acute rejection within the first-year post-transplantation (8). This rate is markedly higher than that observed in solid organ transplants, underscoring the heightened susceptibility of VCAs to rejection (9–12).

Immune rejection in VCA manifests through mechanisms such as T-cell-mediated acute rejection, antibody-mediated rejection, and chronic rejection involving both the innate and adaptive immune system (13, 14). This intricate rejection process involves numerous immune cells, cytokines, chemokines and signaling cascades, collectively contributing to graft failure (13, 15, 16). A deep understanding of the immunopathological mechanisms including the genetic foundation is essential for the development of effective diagnostic and treatment strategies (17, 18). Previous research efforts have explored gene transcript profiles of immune rejection in VCA using methods such as reverse transcription-polymerase chain reaction (RT-PCR) or mRNA NanoString gene expression assays (19–21). However, these techniques have limitations capturing the full spectrum of gene expression, as they are often confined to dozens or hundreds of transcripts, potentially missing crucial genes and pathways. Furthermore, while some studies have characterized the transcriptomes of acute rejection in the muscle of the VCA rat model, few investigations have been conducted in the VCA large animal model, thereby limiting the advancement of this field (17, 18).

To address these knowledge gaps, we turned to the potent tool of RNA sequencing (RNA-Seq) coupled with bioinformatics analysis in a porcine VCA model. This technique is widely recognized for its capacity to comprehensively profile the transcriptome with exceptional sensitivity (22–25). While RNA-Seq has been widely used in solid organ transplantation studies, its application in VCA research, especially in large animal models, remains underutilized (26–29). In our study, we applied RNA-Seq alongside bioinformatics analysis to scrutinize the gene expression signature and underlying biological pathways associated with VCA immune rejection. Additionally, we investigated the gene-level effects of the site-specific, on-demand drug delivery system triglycerol monostearate hydrogel loaded with the tacrolimus (TGMS-TAC), shedding light on its immunosuppressive properties (30). Our research aims to enhance understanding of VCA rejection and to allow for more precise diagnostic and therapeutic approaches, ultimately improving VCA procedure outcomes. Moreover, our open-access RNA sequencing data may serve as a valuable resource for future VCA research endeavors.

## Materials and methods

### Animals and sample preparation

Between 2019-2023, VCA procedures in MHC-mismatched Swiss Landrace pigs as both donors and recipients were performed. These operations were conducted using the heterotopic porcine hindlimb VCA model, previously established and comprehensively documented (31, 32). Briefly, osteo-myo-cutaneous limb allografts, including lymph nodes and skin paddles from the donor pigs, were procured and transplanted into a subcutaneous pocket created in the dorsolateral abdominal wall of the recipient pigs. Vessel anastomosis was performed between the graft and the recipient. The animals were closely monitored until the graft skin displayed extensive epidermolysis, desquamation, and necrosis, serving as the clinical rejection endpoint criterion (corresponding to macroscopic grade III-IV rejection) or until post-operative day (POD) 90. Samples from the graft’s skin and muscle were collected for analysis at the endpoint.

For the analysis performed here, six groups were made: Groups 1, 2, and 3 consisted of skin tissue, while Groups 4, 5, and 6 comprised muscle tissue. The details of the samples are listed in **Supplementary Table 1**.

Healthy controls: Groups 1(skin, n=3) and 4 (muscle, n=3) were composed of healthy donor pigs that underwent no prior manipulation, while all other tissues analyzed were from the rejection groups.

Moderate rejection (TGMS-TAC injection group): In groups 2 (skin, n=3) and 5 (muscle, n=3), the experimental endpoint and tissue collection were reached at POD 90 after receiving repeated intra-graft TGMS-TAC administrations. In these groups, the recipients received treatment with TGMS-TAC containing 7 mg Tacrolimus/mL which was subcutaneously injected in the skin paddle in 0.5 ml depots at a dose of 140 mg per kg graft weight on POD 0, 30, and 60.

Severe rejection (end-stage rejection group): In groups 3 (skin, n=3) and 6 (muscle, n=3), grade III-IV skin rejection was reached after 36, 65, and 56 days, respectively, when tissue was collected. These 3 graft recipients received oral administration of tacrolimus daily for the initial 14 days post-transplantation. In two of the animals, listed as G3a/b and G6a/b, respectively, in **Supplementary Table 1**, oral tacrolimus was followed by intra-graft injections of 1.25 mL/kg-graft rapamycin-loaded *in situ* forming implant loaded with 5 mg of rapamycin per 0.31 mL at multiple sites on POD 15 (33).

### Histological examination

Upon completion of the experiments, tissue samples were obtained from the grafts and fixed in 4% buffered formaldehyde and paraffin-embedded. Further processing followed standard histopathological specimen protocols. Subsequently, these samples were sectioned at a thickness of 3 μm and subjected to hematoxylin and eosin staining for microscopic assessment. Images were acquired using a Pannoramic 250 Flash III slide scanner (3D Histech, Budapest, Hungary). Skin rejection was evaluated blindly and categorized by an experienced pathologist using the Banff 2007 scoring system for skin-containing composite tissue allografts and the swine VCA skin rejection classification (34, 35). Muscle rejection was evaluated blindly and scored for the presence of necrosis and/or atrophy and inflammatory infiltrates, ranging from none (0) to minimal (1), moderate (2) and extensive (3) (36).

### RNA extraction and sequencing

The mRNA transcriptome of the 18 VCA samples was analyzed, including nine skin and nine muscle samples from VCA transplant recipient pigs. The total RNA was extracted according to the instruction manual of the TRIzol Reagent (Life technologies, California, USA). Tissue samples (50-100 mg) were homogenized in 1 ml of TRI reagent and incubated for 5 minutes at the room temperature. Thereafter, 0.2 ml chloroform were added and the mixture agitated for 15 seconds. After an additional 2-3 minutes of incubation, the mixture was centrifuged at 12,000 g for 15 minutes. The RNA was precipitated by mixing the aqueous phase with isopropanol, followed by a 10-minute incubation at the room temperature. After a 10-minute centrifugation at 4°C and 12,000 g, the supernatant was removed, the RNA washed with 75% ethanol and air-dried for 5-10 minutes. The purified RNA was dissolved in RNase-free water, incubated at 55-60°C for 10 minutes and subsequently stored at -70°C. RNA concentration, purity, and integrity were measured using the NanoDrop 2000 and the Agilent Bioanalyzer 2100 systems.

For RNA sequencing, we used 1 μg of RNA per sample. Sequencing libraries were created with the Hieff NGS Ultima Dual-mode mRNA Library Prep Kit for Illumina, including index codes for sample identification. This involved mRNA purification, first and second-strand cDNA synthesis, blunt-end conversion, and adenylation of DNA fragment 3’ ends. Then NEBNext adaptors with hairpin loop structures for hybridization were ligated. Library fragments were purified using the AMPure XP system, followed by a 15-minute USER Enzyme treatment at 37°C then 5 minutes at 95°C before PCR. PCR was performed with Phusion High-Fidelity DNA polymerase, Universal PCR primers and Index (X) Primer. PCR products were purified with the AMPure XP system, and the library quality assessed with the Agilent Bioanalyzer 2100. Sequencing was performed on an Illumina NovaSeq platform, generating 150 bp paired-end reads as per the manufacturer’s instructions.

The RNA integrity number (RIN value) averaged 7.1 ± 1.2, which qualified for mRNA transcriptome sequencing (**Supplementary Table 1**). In this study, a total of 27,601 genes were identified, which included 6,924 new genes (**Supplementary Table 2**). To assess the dispersion of gene expression within samples and compare overall expression among samples, box plots were utilized. Spearman’s correlation coefficient (R) to evaluate the reproducibility of biological replicates was applied. A higher R² value, closer to 1, indicates better reproducibility between two samples (**Supplementary Figure 1**, **Supplementary Table 3**). To gain insight into the relationships among samples, principal component analysis (PCA) based on the Fragments Per Kilobase of Transcript Per Million Fragments Mapped (FPKM) values of each sample was performed. The PCA allowed us to visualize the similarity among samples by reducing dimensionality into two or three principal components (**Supplementary Figure 1**). Among our 18 samples, all except G5a and G6a, demonstrated good reproducibility and similarity within the same group in terms of gene expression. This observation aligned with the findings of the pathological evaluation.

### Data processing and bioinformatics analysis

The initial processing of raw data in fastq format was performed using custom Perl scripts. This process involved the removal of reads containing adapters, poly-N sequences, and low-quality reads. Simultaneously, quality metrics, including Q20, Q30, GC-content, and sequence duplication levels to ensure the cleanliness of the data were assessed. To prepare the data for analysis, we further removed adapter sequences and low-quality reads. All subsequent analyses were conducted using this high-quality, clean data. In this study, a total of 18 samples were processed for transcriptome sequencing, generating 123.51 GB of clean data. Each sample yielded a minimum of 5.91 GB of clean data, with a minimum of 93.48% of clean data achieving a quality score of Q30. Clean reads of each sample were mapped to Sus_scrofa.GCF_000003025.6.genome.fa, with mapping ratios ranging from 83.88% to 96.59%.

Gene expression levels were quantified using FPKM. For functional annotation, various databases such as Nr, Pfam, KOG/COG, Swiss-Prot, and KO were utilized. Additionally, cell type abundance was estimated using the Cibersort tool, leveraging normalized bulk RNA-Seq expression data (FPKM) as input, and relative cell type abundance across all samples was visually depicted (37–39). Differential expression genes (DEGs) analysis was conducted through DESeq2, which employs a negative binomial distribution model to identify differential expression in digital gene expression data. Genes with an adjusted P-value < 0.01 and a fold change ≥ 1.5, as determined by DESeq2, were classified as differentially expressed. For a deeper understanding of the differentially expressed genes, Gene Ontology (GO) enrichment analysis, including biological processes, cellular components, and molecular functions, was performed. Kyoto Encyclopedia of Genes and Genomes (KEGG) pathway analysis allowed us to comprehend high-level biological system functions. Gene set enrichment analysis (GSEA) was also conducted.

### Immunofluorescence

To prepare samples, 6 μm sections of tissue frozen in Tissue-Tek optimal cutting temperature compound (OCT, Sakura) were obtained using the Leica Cryostat CM1950. These sections were stained with specific antibodies against CD31 (R&D Systems, Ref. MAB33871), IL1β (Proteintech, 16806-1-AP), and Caspase-3 (Thermo Fisher, MA1-91637). Cell nuclei were visualized using DAPI staining (Sigma, Ref. 10236276001). Images were acquired with an LSM Zeiss 980 confocal microscope and processed using ImageJ software.

### Transmission electron microscope

Ultrastructural analysis of the rejected tissue was conducted using transmission electron microscopy (TEM). Fresh samples of skin and muscle were fixed in 4% formaldehyde and then processed, including demineralization, fixation, staining, and embedding in EPON 812 (TAAB Laboratories Equipment, Berks, United Kingdom). Ultrathin sections (70 to 80 nm) were examined with a TEM (H-7600; Hitachi, Tokyo, Japan). Diagnosis and evaluation of the rejected tissues were performed by an electron microscopy pathologist.

### Statistical analysis

Quantitative data were expressed as mean ± SD. We used DESeq2 to determine fold changes. DESeq2 fits a negative binomial model of the sequence data and derives probability values for differential expression using an exact test. P values were adjusted using the Benjamini and Hochberg method to control the false discovery rate. P< 0.05 was considered statistically significant.

## Results

### Histological assessment of VCA rejection samples

**Figure 1:**
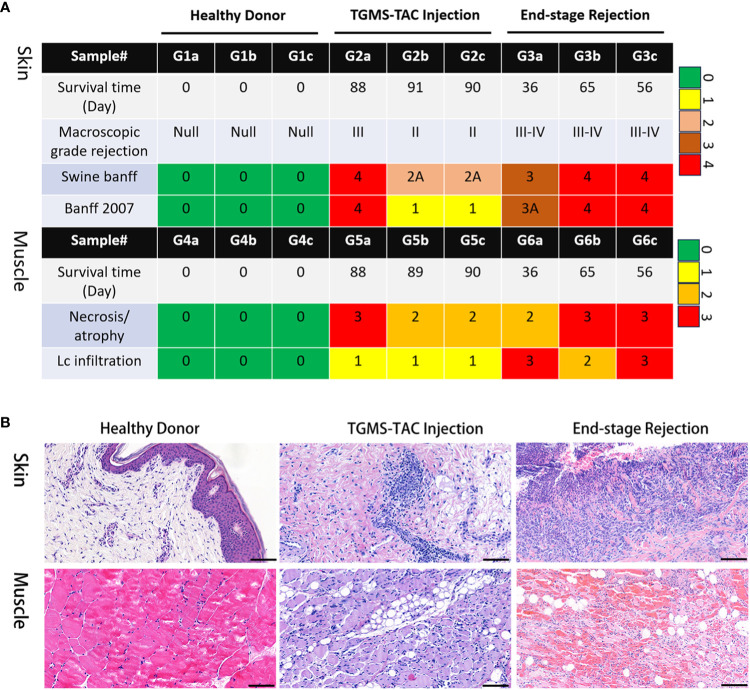
Histological evaluation of VCA rejection. **(A)** Pathological scoring of VCA rejection samples, with TGMS-TAC treated group exhibiting lower scores in both skin and muscle tissues in comparison to the untreated rejection group. **(B)** Representative images showcasing the pathological features of rejection in skin and muscle tissues.

Both skin and muscle samples from the healthy control group, displayed normal histological findings. However, the TGMS-TAC injection and end-stage groups represent distinct stages of rejection, with the former displaying moderate rejection and the latter showing severe rejection. Except for one recipient, lower pathological scores in both skin and muscle of the TGMS-TAC injection group were observed compared to the end-stage rejection group (**Figure 1A**). The pathological features of rejection in the skin and muscle tissues of the end-stage rejection groups included full-thickness epidermal necrosis, lymphocytic cell infiltration, hemorrhage, and perivascular-accentuated inflammation. In addition, muscle tissue in end-stage rejection group exhibited prominent atrophy and fatty replacement in comparison to the muscle tissue of the TGMS-TAC injection group (**Figure 1B**; **Supplementary Table 1**).

### Differentially expressed genes and pathway analysis comparing non-rejection and rejection

**Figure 2:**
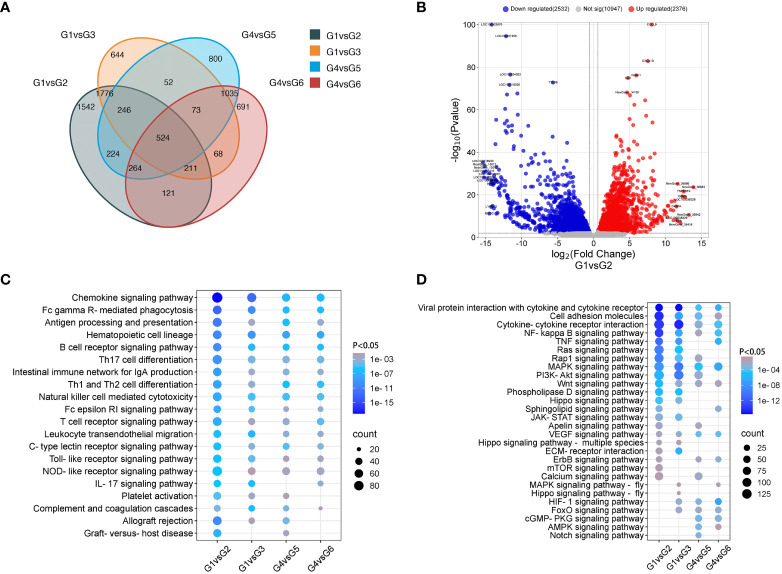
Comparative analysis of gene expression in non-rejection and rejection. **(A)** Venn diagram depicting the overlap of differentially expressed genes among the groups. **(B)** Volcano plot of differentially expressed genes between groups 1 and 2. **(C, D)** Enrichment of immune system and signal transduction pathways in KEGG analysis.

To understand the transcriptomic profiles of the rejected VCA grafts, a comprehensive analysis of gene expression differences between rejection and non-rejection groups in both skin and muscle samples from VCA grafts was conducted. Using DEGs analysis, the changes in the gene expression of skin and muscle samples of TGMS-TAC injection and end-stage rejection groups in comparison to their respective control were analyzed. We observed several DEGs that are specific to each group as well as some shared DEGs among groups (**Figure 2A**). For instance, in the skin of the TGMS-TAC injection group compared to healthy skin, 4908 genes exhibited differential expression, with 2376 genes upregulated and 2532 genes downregulated (**Figure 2B**). GO and KEGG analysis highlighted the significance of immune and inflammatory responses, as well as antigen processing and presentation (**Supplementary Figure 2**). Pathway enrichment analysis results revealed that the DEGs are associated with several pathways including chemokine signaling, viral protein interactions with cytokines and cytokine receptors, and TNF signaling. Similarly, the skin of the end-stage rejection group showed differential expression in 3594 genes, with 1689 genes upregulated and 1905 genes downregulated compared to healthy skin (**Supplementary Figure 3**). The most strongly associated biological processes involved in this group were immune response, keratinocyte differentiation and epidermis development. Pathway analysis emphasized the involvement of chemokine signaling, viral protein interactions as well as MAPK and PI3K-Akt signaling pathways.

In muscle samples, compared to the healthy control group, the TGMS-TAC injection group exhibited differential expression in 3218 genes, with 1598 genes upregulated and 1620 genes downregulated (**Supplementary Figure 4**). Here, immune response and inflammatory response were the most amplified biological processes. The chemokine signaling pathway was noted as the most involved pathway. In the muscle tissue of the end-stage rejection group, 2987 genes displayed differential expression, with 1478 genes upregulated and 1509 genes downregulated compared with the healthy control group (**Supplementary Figure 5**). Immune response, inflammatory response and mitochondrial electron transport featured prominently in GO analysis, while the MAPK signaling pathway emerged as a significantly enriched pathway. Detailed gene lists can be found in **Supplementary Tables 2**, **3**.

Furthermore, pathway analysis of the immune system and signal transduction indicates the enrichment and upregulation of immune-related pathways, such as chemokine signaling, antigen processing and presentation, Th1 and Th2 cell differentiation and T cell receptor signaling in the skin and muscle of the TGMS-TAC injection and end-stage rejection groups (**Figures 2C, D**). Compared with muscle, the skin of the TGMS-TAC injection and end-stage rejection groups showed more severe rejection with higher expression of immune rejection related genes. The KEGG pathway (mmu04060) for cytokine-cytokine receptor interaction in control and rejection group comparisons has been analyzed (**Supplementary Figure 6**). Consistently, the common top upregulated 20 DEGs among group comparisons included GZMA, IL-1β, and TNFRSF4, which are linked to immune cell regulation in the context of immune rejection (**Supplementary Table 4**). Immunofluorescence analysis confirmed the upregulation of IL-1β in skin and muscle of the end-stage rejection groups compared to healthy tissues (**Supplementary Figure 7**).

### Expression of immune-relevant genes in VCA rejection

**Figure 3:**
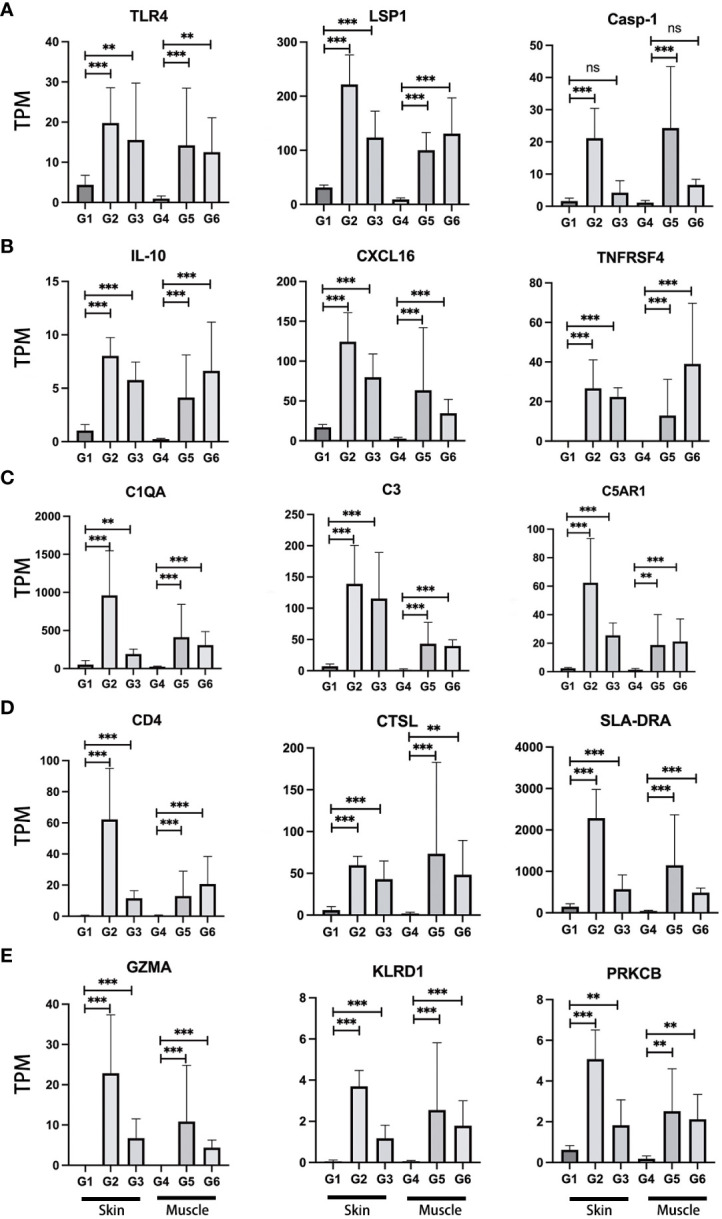
Expression profiles of representative immune-relevant genes. A range of genes implicated in immune rejection demonstrated increased activity. These genes are associated with various immune system components and responses, including **(A)** pattern recognition receptors (TLR4), damage/danger associated molecular patterns (LSP1), Caspase (Casp-1); **(B)** cytokines (IL-10), chemokines (CXCL16), tumor necrosis factor (TNFRSF4); **(C)** complement (C1QA, C3, C5AR1); **(D)** antigen processing and presentation (CD4, CTSL, SLA-DRA); **(E)** cytotoxicity (GZMA, KLRD1, PRKCB). For a detailed list of the immune-relevant genes, please refer to **Supplementary Table 5**. TPM: Transcripts Per Million. G1-6: experimental groups. G1-3: skin, G4-6: muscle tissue. **P<0.01, ***P<0.001, and 'ns' indicates not significant.

To evaluate the expression of previously described immune-relevant genes in VCA rejection, a comprehensive analysis of genes related to the immune response was performed (**Supplementary Table 5**) (40–44). First, we focused on investigating the expression of genes associated with pattern recognition receptors (PRRs), which play a central role in the function of the innate immune system. Among these receptors, Toll-like receptors (TLRs) and NOD-like receptors (NLRs) serve as distinct sensors, specifically designed to recognize these patterns. In our analysis, significant differences in gene expression for various members of the NLR and TLR families, including NLRP1, NLRP3, NLRP12, TLR1, TLR2, TLR4, TLR6, TLR7, and TLR8 were found (**Figure 3A**; **Supplementary Table 5**). These differences were observed in both skin and muscle of the TGMS-TAC injection and end-stage rejection groups when compared to healthy controls, indicating that altered innate immune responses contribute to VCA graft rejection. Next, we explored the expression of genes related to damage/danger-associated molecular patterns (DAMPs), which act as ligands for PRRs. Specifically, up-regulation in the expression of genes such as DNAJA1, HIF1A, HMGB1, HMGB2, HMOX1, HSP90B1, HSPA5, S100A12, S100A11, LSP1, BASP1 and SYK in the skin of the TGMS-TAC injection and end-stage rejection groups were observed. In muscle tissue of the TGMS-TAC injection and end-stage rejection groups, up-regulation in the expression of HMGB2, S100A11, LSP1, BASP1 and SYK was found. These findings indicate that activation of various PRRs, which recognize and respond to distinct molecular patterns, ultimately contribute to the immune rejection in VCA.

The activation of PRRs triggers a cascade of events, culminating in the expression of essential immune mediators, including cytokines, chemokines, interferons (IFNs) and tumor necrosis factor (TNF). The significant up-regulation of genes within the caspase family, notably Casp-1, Casp-3, Casp-7, Casp-8, and Casp-10, were shown in the skin of the TGMS-TAC injection and end-stage rejection groups when compared to healthy skin (**Figure 3A**; **Supplementary Table 5**). Additionally, Casp-1 and Casp-6 displayed remarkable up-regulation in the muscle of the end-stage rejection group in comparison to healthy muscle. IFNs are pivotal in immune regulation and up-regulation including IFNGR1, IFNGR2, IFNAR1 and IFNAR2 in skin of the end-stage rejection group points towards an active immune response in this tissue. Within the TNF family, a series of genes, including TNF and various TNF-related factors, exhibited significant up-regulation in skin of the TGMS-TAC injection and end-stage rejection groups (**Figure 3A**; **Supplementary Table 5**). Various chemokines, including CXCL2, CXCL8, CXCL9, CXCL10, CXCL11 and CXCL16 were up-regulated in skin and muscle of the TGMS-TAC injection and end-stage rejection groups compared to healthy skin and muscle (**Figure 3B**; **Supplementary Table 5**). These chemokines are essential for recruiting immune cells to sites of inflammation and injury, underlining their role in the mobilization of immune cells to the rejection site and the establishment of an inflammatory microenvironment. Moreover, cytokines are central to immune responses and inflammation regulation. Significant up-regulations in inflammation-related cytokines such as IL-1α, IL-1β, IL-6, IL-10, IL-12, IL-13, IL-17RA, IL-18 and IL-33 in skin and muscle of the TGMS-TAC injection and end-stage rejection groups were observed (**Figure 3B**; **Supplementary Table 5**). These cytokines play vital roles in regulating inflammatory and immune responses and their up-regulation in the context of immune rejection signifies the induction of a robust and well-coordinated immune response.

Next, the involvement of genes related to the complement system was examined (**Figure 3C**; **Supplementary Table 5**). An abundance of key activators of the classical complement pathway, including C1QA, C1QB, C1Q, C1R and C1S, were notably higher in the skin and muscle of the TGMS-TAC injection and end-stage rejection groups when compared to healthy skin and muscle. The mRNA level of C3, which is activated in all three complement pathways, was significantly elevated in skin and muscle of the TGMS-TAC injection and end-stage rejection groups. Additionally, the receptor for C3, C3AR1, exhibited a substantial increase in its expression. C3 convertase proteins involved in the classical pathway, C4A and C2, both showed significantly higher expression levels in skin and muscle of the TGMS-TAC injection and end-stage rejection groups. A protein related to alternative pathway complement activation, CFB, was also higher expressed in these groups. The key lectin pathway activating gene, MASP1, exhibited differences only in the skin of the TGMS-TAC injection and end-stage rejection groups, but not in muscle tissue. Moreover, the abundance of C3 receptor (C3AR1), C5 receptor (C5AR1), as well as VSIG4, demonstrated higher expression levels in both skin and muscle of the TGMS-TAC injection and end-stage rejection groups. The terminal complement component C9 showed no significant difference in expression within the skin tissues, but it was elevated in muscle tissue of the TGMS-TAC rejection group.

Our examination of genes associated with antigen processing and presentation, a critical aspect of immune responses, revealed a unique upregulation of several genes such as CD4, CTSL and SLA (the pig analog of the human histocompatibility antigen HLA) in both skin and muscle of the TGMS-TAC injection and end-stage rejection groups, distinguishing them from healthy skin and muscle. These genes play a pivotal role in the recognition and presentation of antigens to the immune system (**Figure 3D**; **Supplementary Table 5**). Additionally, genes related to cytotoxicity such as GZMA, KLRD1 and PRKCB were also found to be upregulated in the skin and muscle of the TGMS-TAC injection and end-stage rejection groups, compared to healthy skin and muscle (**Figure 3E**; **Supplementary Table 5**). These genes are closely associated with cytotoxic immune responses and the elimination of target cells during rejection.

A comprehensive analysis of gene expression related to critical cellular processes, including cellular oxidative stress, glycolysis and gluconeogenesis, endoplasmic reticulum stress as well as DNA repair was conducted. Detailed results and gene expression profiles for these processes are shown in **Supplementary Table 5**.

### Enrichment of immune cell types

**Figure 4:**
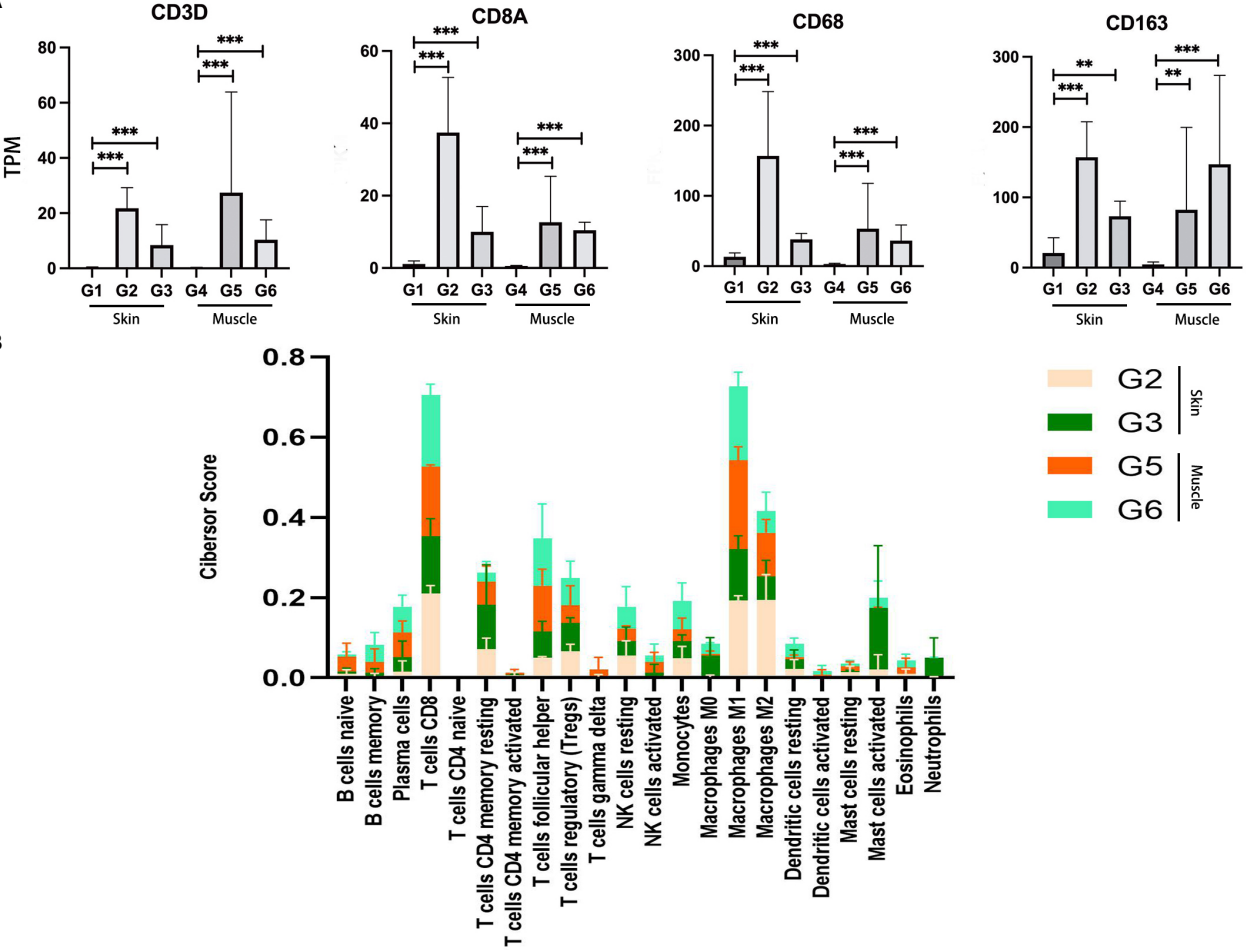
Enrichment analysis of immune cell types in VCA rejection tissue. **(A)** Increased expression of T cell (CD3D and CD8A) and macrophage (CD68 and CD163) related genes in the rejection group. **(B)** Immune cell type scores derived from gene expression data in the rejection tissue. **P<0.01, *** P<0.001.

To analyze the involvement of immune cells in VCA rejection, the expression of genes such as CD3D and CD8A was analyzed, indicative of T cells, along with CD68 and CD163, associated with macrophages. These genes were found to have higher expression levels in skin and muscle of the TGMS-TAC injection and end-stage rejection groups compared to the healthy skin and muscle groups (**Figure 4A**). Further insight into the expression of additional CD molecular markers, such as CD40 and CD86, were also investigated (**Supplementary Table 5**), revealing an overall upregulation of CD biomarkers of immune cells in VCA tissues. To gain a deeper understanding of the immune cell composition within the VCA rejection tissue, cell-type enrichment analysis was performed using the Cibersort tool based on gene expression data for 22 immune cell types. The analysis indicated that skin and muscle of the end-stage rejection groups exhibited higher immune scores compared to healthy skin and muscle (**Supplementary Figure 8**). Among the 22 immune cell types in skin and muscle of the TGMS-TAC injection and end-stage rejection groups, macrophages M1, T cells CD8, and macrophages M2 were identified as the highest scored cell types, with follicular helper T cells, regulatory T cells, and resting CD4 T memory cells also being prominent immune cell populations within the VCA tissue (**Figure 4B**).

### Cell death in VCA rejection

**Figure 5:**
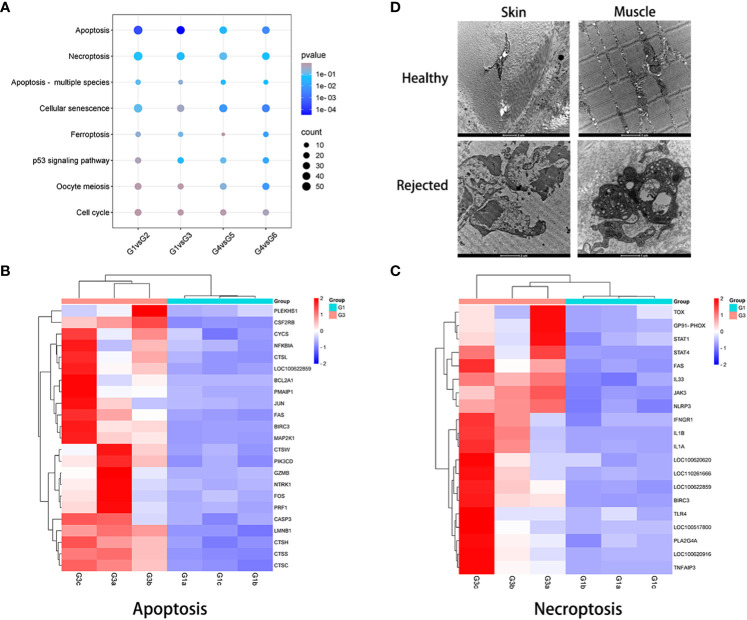
Examination of cell death in VCA rejection. **(A)** KEGG pathway analysis related to cell growth and death between non-rejection and rejection groups. **(B, C)** Expression patterns of genes involved in apoptosis and necroptosis in skin samples from groups 1 and 3. **(D)** Transmission electron microscopy evaluation of rejected VCA tissues, revealing cell necrosis and extensive collagen fiber dissolution in both skin and muscle compared with healthy tissue.

To investigate cell death in VCA during immune rejection, pathways related to cell growth and death were explored through KEGG enrichment analysis. When compared to healthy skin and muscle, significant differences in apoptosis were observed across skin and muscle of the TGMS-TAC injection and end-stage rejection groups. Necroptosis exhibited significant differences in the skin of the end-stage rejection group when compared to healthy skin, and ferroptosis showed substantial differences in the muscle of the end-stage rejection group compared to healthy muscle (**Figure 5A**). The DEGs associated with apoptosis and necroptosis, as indicated by the heatmap, were specifically analyzed for each recipient in the skin of the end-stage rejection group compared to healthy skin. Genes involved in apoptosis and necroptosis, such as FAS, CASP3, IL1α, IL1β, IL33, NLRP3 and TOX exhibited a significant upregulation (**Figures 5B, C**).

To further validate these findings, immunofluorescence analysis confirmed the upregulation of caspase-3 in skin and muscle of the TGMS-TAC injection and end-stage rejection groups compared to healthy skin and muscle (**Supplementary Figure 9**). Interestingly, Transmission Electron Microscopy (TEM) examination revealed prominent manifestations of cell necrosis and widespread collagen fiber dissolution in both skin and muscle of end-stage rejection groups compared with healthy control groups (**Figure 5D**). These were characterized by the absence of cell membranes, nuclear decomposition as well as shrinkage and disintegration of cell structures.

### The effect of TGMS-TAC on muscle by GSEA analysis

**Figure 6:**
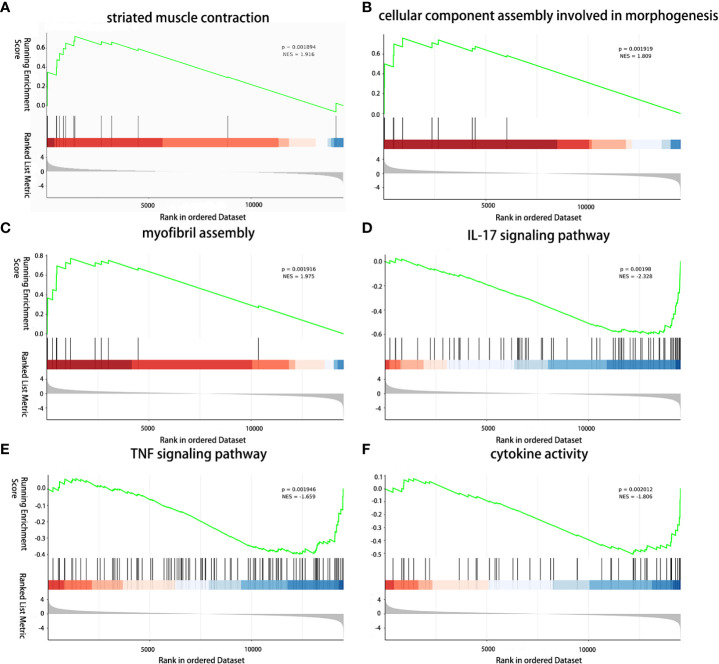
Immunomodulatory effect of TGMS-TAC on the muscle transcriptome. **(A)** GSEA analysis reveals upregulation of striated muscle contraction, **(B)** cellular component assembly involved in morphogenesis, and **(C)** myofibril assembly, and **(D)** downregulation of IL-17 signaling pathway, **(E)** TNF signaling pathway, and **(F)** cytokine activity between TGMS-TAC injection group and end-stage rejection group.

The impact of TGMS-TAC on muscle tissue at the transcriptome level was further investigated based on histological differences and gene expression in muscle of the TGMS-TAC injection group and muscle of end-stage rejection group. To discern the immunosuppressive effects of TGMS-TAC, GSEA was employed to evaluate gene signatures associated with immune rejection and inflammation (**Supplementary Table 6**). The upregulated gene signature was found to be enriched in processes related to striated muscle contraction (P<0.01, NES=1.916), cellular component assembly involved in morphogenesis (P<0.01, NES=1.809) and myofibril assembly (P<0.01, NES=1.905), implying a better preservation of muscle and function in the muscle of the TGMS-TAC injection group and suggesting a protective effect on the muscle tissue by TGMS-TAC compared to the muscle of the end-stage rejection group (**Figure 6**). In contrast, the downregulated gene signature was enriched in pathways associated with IL-17 signaling (P<0.01, NES=-2.328), TNF signaling (P<0.01, NES=-1.659) and cytokine activity (P<0.01, NES=-1.806) in the group that received TGMS-TAC. This suggests that rejection and inflammation in muscle were less activated in the presence of TGMS-TAC, aligning with the pathological results.

## Discussion

Aiming to better understand the immune rejection mechanisms in VCA, we employed bulk-RNA sequencing complemented by advanced bioinformatics analysis (45, 46). The application of RNA-Seq in VCA research, especially in a large animal model, represents a significant advancement in the field (29). By integrating this technology with comprehensive bioinformatics analysis, we characterized the transcriptomic profile of immune rejection in the composite skin and muscle tissues in VCA, shedding light on the intricate processes underlying graft rejection. Understanding the dynamics and the molecular pathways involved in graft rejection may help to develop strategies to tackle the high rates of acute rejection observed in VCA.

In our study, we conducted heterotopic vascularized composite graft allotransplantation in a preclinical large animal model. Samples were collected from skin and muscle of grafts which were fully rejected (end-stage rejection groups), as well as from grafts which were treated with TGMS-TAC injection (TGMS-TAC treatment groups). The latter grafts experienced various degrees of rejection, generally pathologically milder than end stage groups. Healthy skin and muscle tissues from the graft donors were used as control groups. The groups were primarily categorized based on the pathological degree of tissue rejection, a common practice in clinical transcript-related studies (19, 40). The sequencing results exhibited overall consistency and high repeatability, indicating that the pathological rejection grading primarily influences transcriptional differences. Inclusion of both TGMS-TAC injection and end-stage groups, due to their varying degree of rejection, was aimed at comprehensively capturing gene changes at different stages of immune rejection in VCA samples.

Our results showed substantial increases in the expression of genes related to PRRs in VCA, which are central to the innate immune responses and initiation of further adaptive immune responses (47). Our findings also align with prior research, highlighting the predominant role of DAMPs in the innate immune system’s PRR signaling in kidney rejection (40). Notably, we observed significant changes in the expression of members from the nucleotide binding NLR and TLR families, indicating recognition of distinct molecular patterns as a critical early step in immune responses to VCA tissues. Additionally, we detected the upregulation of several DAMP-related genes in rejected tissues, including HMGB1 (a well-characterized nuclear protein DAMP involved in ischemia-reperfusion injury), and HIF1A (a master transcriptional regulator in response to hypoxia). Targeting the processes regulated by these DAMPs might serve as a possible therapeutic target to early steps of graft rejection in VCA, which are caused by the innate immune system and set the stage for the subsequent, deleterious adaptive immune response. We currently have rather good tools to prevent the latter, but the availability of specific drugs to prevent the initial innate immune activation is still limited or they are in early preclinical development.

Activation of PRRs triggers a cascade of events leading to the expression of pivotal immune mediators. Our study highlights the upregulation of various cytokines, chemokines, interferons, and TNF family members in VCA rejection in line with previous findings (16, 20). We observed significant upregulation of cytokines such as IL-1β, IL-6, IL-33 and CXCL8, indicating a robust and coordinated immune response in rejected grafts by recruiting immune cells to the rejection site and establishment of an inflammatory microenvironment. We also identified a unique upregulation of numerous antigen processing and presentation genes in this swine VCA model, indicating active recognition and presentation of antigens to the immune system. Moreover, genes associated with cytotoxicity such as GZMA, GZMB and PRF1 as well as pathways including T cell receptor signaling and natural killer cell-mediated cytotoxicity were also upregulated, reflecting the involvement of cytotoxic immune responses in VCA graft rejection, which is also supported by a previous study (19).

Our analysis further uncovered distinct variations in genes and pathways associated with the complement system. The classical complement pathway appears to be significantly activated during VCA rejection, as evidenced by the upregulation of gene transcription for crucial complement components like C1, C2, C3 and C5. In addition, our data also suggest the involvement of the lectin and alternative pathways, suggesting a contribution of all three complement activation pathways to VCA rejection. Moreover, complement activation in VCA contributes to the development of the inflammatory microenvironment and further recruitment of immune cells to the VCA site. As targeted complement inhibition has been shown to ameliorate graft injury in VCA, this could serve as a promising therapeutic strategy (48).

Our immune cell-type enrichment analysis indicated that CD8 T cells, macrophages M1, and macrophages M2 were the most prominent cell types in VCA rejection. These immune cell populations play key roles in immune responses and inflammation in the graft. Our findings further confirm the role of T cells in mediating tissue injury, along with increased numbers of proliferative T cells expressing markers of antigen-specific activation, CD8+ and CD4+ T cells and T cells expressing perforin and granzyme in VCA (15, 19). In addition to T cells, macrophages, which are phagocytic cells capable of engulfing cellular debris, apoptotic cells and pathogens during rejection, were enriched along with other innate immune cell types like neutrophils and NK cells. This highlights the cellular diversity and complexity of the immune response in VCA rejection (15).

Upregulation of genes such as FAS, Casp-3 and NLRP3 in VCA tissues highlights the role of apoptosis, necroptosis and ferroptosis in rejection of tissues (49). Notably, the differential expression of genes involved in these cell death pathways align with the observed histological features. TEM further highlighted widespread cell necrosis and collagen fiber dissolution, supporting the notion of extensive cell death in VCA rejection. These results are consistent with the coexistence of multiple cell death types observed in solid organ rejections (40, 50).

By enabling controlled and localized drug delivery, TGMS-TAC holds the potential to reduce systemic immunosuppression-related side effects, offering a more precise and effective means of managing rejection (30, 51–54). To provides a holistic view of pathway-level changes and mitigate errors arising from individual gene expression levels, GSEA analysis was conducted. Through this method, our study further revealed that TGMS-TAC treatment in VCA is associated with a protective effect on muscle tissue, promoting muscle integrity and reducing inflammation on the histological and transcriptional level. The downregulation of pathways related to IL-17 and TNF-signaling further supports the immunosuppressive properties of TGMS-TAC in VCA inflammation. Despite TGMS-TAC treatment, grafts still exhibited some degree of rejection when compared to healthy tissues, as evidenced by increased expression of immune rejection-associated genes. Samples from the TGMS-TAC group, clinically and pathologically displaying up to grade 2-3 rejection, demonstrated a robust immune rejection process on the transcriptional level consistent with prior studies (19). This heightened expression of immune rejection-related genes in the TGMS-TAC group suggests a vigorous immune response, as gene transcription often precedes observable biological manifestations. In contrast, end-stage samples displayed advanced immune rejection processes, primarily resulting in necrosis. Consequently, some molecular expressions of immune rejection-related genes were higher in the TGMS-TAC group compared to the end-stage group. These findings underscore the dynamic nature of immune rejection processes, highlighting the significance of considering both timing and context in gene expression analyses.

VCA encompasses the transplantation of multiple tissues, such as skin, muscle, and cartilage, setting it apart from organ transplantation. Long-term survival and functional reconstruction in VCA are intricately linked to the diverse nature of these tissues. Our study conducted a comparative analysis of rejection levels between skin and muscle within the same graft, revealing skin’s heightened susceptibility to rejection compared to muscle on the transcriptional and histological level, attributed to its high immunogenicity within the VCA context (13, 36). CD4+ and CD8+ T cells immune responses have been described as the main player in skin graft rejection (55, 56). Achieving an immunosuppressive effect in skin is particularly challenging, and different strategies to ameliorate the immune rejection of skin will therefore be needed to increase the long-term success of VCA (57, 58).

Early recognition of rejection is crucial for graft survival. In addition to the pathological changes, our study identified the top 20 genes involved in VCA rejection. RNA transcriptome analysis provides early biological information of protein translation (45, 46). Increased levels of GZMA, IL-1β and TNFRSF4 could potentially serve as biomarkers for early rejection diagnosis. However, this will need to be confirmed in studies in which an analysis of samples at different timepoints after transplantation will be performed.

In comparison to organ transplantation, research on VCA, especially in large animal models, has been relatively underexplored in the realm of gene sequencing. Our study initially utilized bulk RNA analysis to investigate transcriptome profiling in a porcine VCA model. Similar to findings in organ transplantation, higher levels of DAMPs resulting from cellular oxidative stress, endoplasmic reticulum stress, and DNA repair serve as ligands for signaling through PRRs in the rejection process (40, 41). The increased expression of mRNAs encoding cytokines, chemokines, interferons, caspases, and complement factors reflected similarities between VCA and organ transplantation. A higher abundance of T cells in rejected VCA tissues through cell-type-enrichment analysis implied the heightened susceptibility of VCAs to rejection compared to organ transplants. Additionally, similar to organ transplantation, pathways related to apoptosis and necroptosis were significantly enriched in rejected VCA tissues (40). While our study provides a comprehensive gene-level landscape for VCA, further experimental validation is required to elucidate the specific roles and functions of individual pathways and related proteins, such as necroptosis in VCA.

Several limitations should be acknowledged in this study. Firstly, due to the difficulty in establishing large animal models with long-term follow-up observations, this study was conducted with a limited number of samples from large animal experiments, leading to variations in the extent of rejection among the samples and therefore differences in the generated gene analysis data. Additionally, the absence of a completely untreated control group represents a weakness in our study design. The different treatment modalities in the rejection groups may have influenced gene expression. Furthermore, our study only analyzed skin and muscle tissues, lacking examination of other vascularized tissues such as bone and cartilage. Moreover, single-cell RNA sequencing has emerged as a powerful method for elucidating gene expression patterns and intercellular signaling networks at a single-cell level (59, 60). This technology enables the discovery of new cell subtypes and differentiation between immune cells originating from the donor and recipient within the graft. Accumulating evidence underscores its effectiveness in assessing immune responses in organ transplantation, suggesting promising applications in the realm of VCA (61, 62).

In summary, our study sheds an initial extensive view on the genetic signature within the porcine VCA model. We underscore the critical necessity of comprehending the molecular landscape of immune rejection mechanisms. Our exploration spans the transcriptome level, meticulously dissecting the progression from innate immunity activation to the pivotal stages of antigen recognition, cytotoxic rejection, and eventual cell death observed in VCA rejection tissues. This research serves to deepen our understanding of the intricate mechanisms underlying graft rejection and holds promise for refining diagnostic and therapeutic strategies, ultimately enhancing the success and long-term viability of VCA procedures.

## Data availability statement

The datasets presented in this study can be found in online repositories. The names of the repository/repositories and accession number(s) can be found below:E-MTAB-13722 (ArrayExpress; https://www.ebi.ac.uk/biostudies/arrayexpress/studies/E-MTAB-13722?key=ed5d6f23-f770-4cb4-85e4-22c7b95408f4)”.

## Ethics statement

The animal study was approved by the Veterinary Office of the Canton of Bern, Switzerland, (approval number: BE48/19). The study was conducted in accordance with the local legislation and institutional requirements.

## Author contributions

LZ: Conceptualization, Data curation, Formal analysis, Investigation, Methodology, Software, Writing – original draft, Writing – review & editing. IA: Investigation, Methodology, Writing – review & editing. AH: Data curation, Validation, Writing – review & editing. YB: Investigation, Methodology, Writing – review & editing. CZ: Methodology, Writing – review & editing. IL: Investigation, Methodology, Validation, Writing – review & editing. SH: Investigation, Methodology, Project administration, Writing – review & editing. MC: Resources, Supervision, Writing – review & editing. RR: Investigation, Methodology, Resources, Supervision, Validation, Writing – review & editing. MG: Methodology, Supervision, Writing – original draft, Writing – review & editing. RO: Funding acquisition, Resources, Writing – original draft, Writing – review & editing.
