# Supplementary material for: Transcriptome profiling of immune rejection mechanisms in a porcine vascularized composite allotransplantation model
Source: Front Immunol. 2024 May 21;15:1390163. doi: 10.3389/fimmu.2024.1390163 (PMC11151749; doi:10.3389/fimmu.2024.1390163)
Supplement: Supplementary file 7 [file DataSheet_1.pdf]

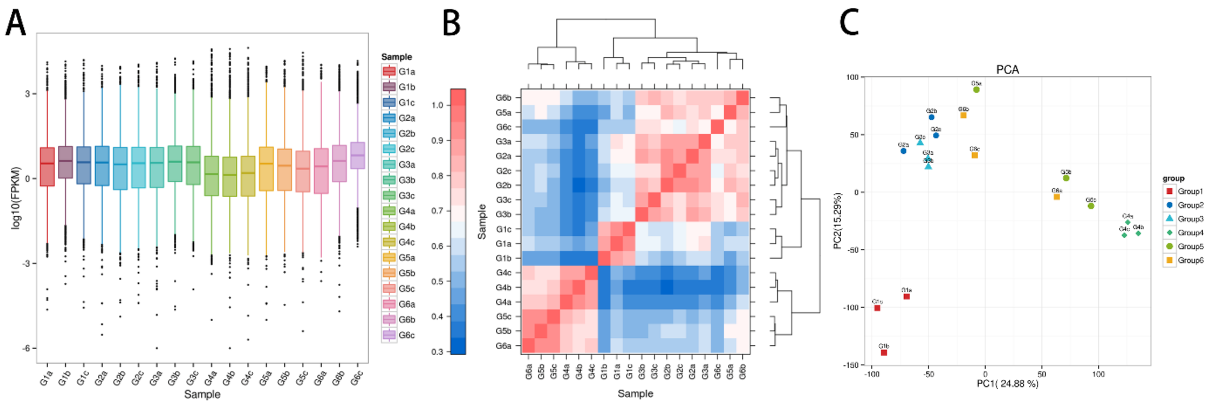

Supplementary Figure 1. Assessment of gene expression dispersion and reproducibility. (A) Box plots were generated using FPKM values to assess gene expression dispersion within samples and compare overall expression across samples. (B) Spearman's correlation coefficient (R) was employed to gauge the reproducibility of biological replicates. (C) Principal component analysis (PCA) was conducted using FPKM values to analyze sample relationships. Within the dataset comprising 18 samples, consistent reproducibility and similarity were observed among samples within the same group, particularly in terms of gene expression correlation with pathological rejection grading.

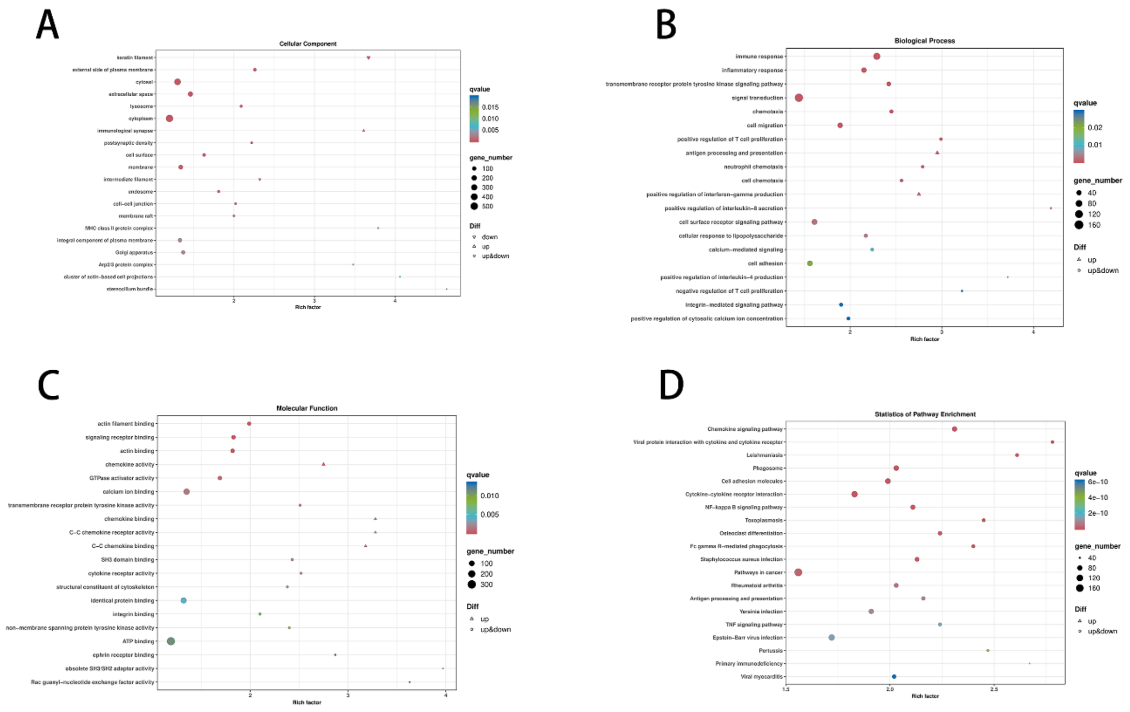

Supplementary Figure 2. Analysis of differentially expressed genes and related pathways between groups 1 and 2. (A) Cellular component in GO analysis of the differentially expressed genes. (B) Biological process in GO analysis of the differentially expressed genes. (C) Molecular function in GO analysis of the differentially expressed genes. (D) Statistics of pathway enrichment in KEGG analysis of the differentially expressed genes.

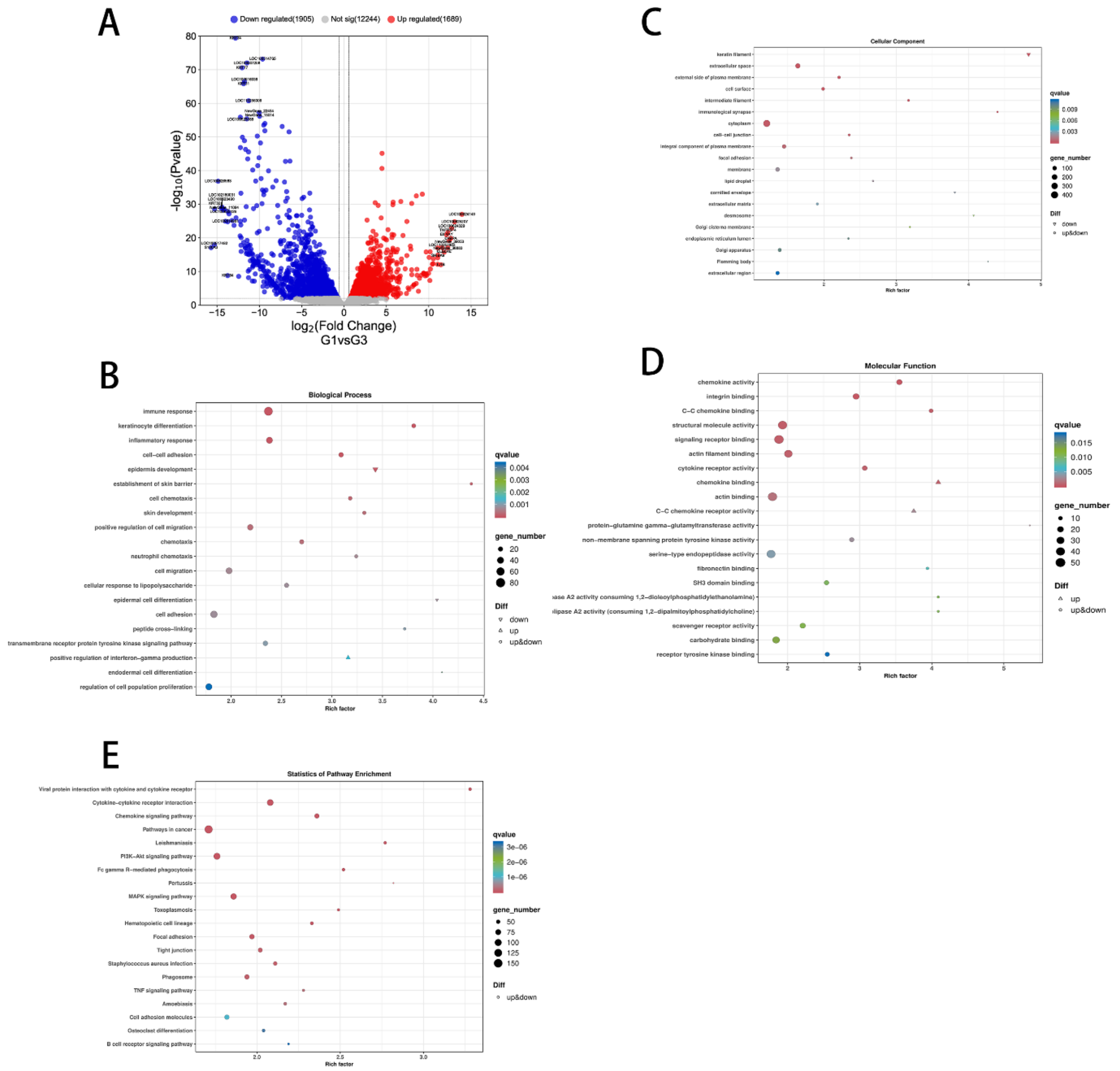

Supplementary Figure 3. Analysis of differentially expressed genes and related pathways between groups 1 and 3. (A) The skin of the end-stage rejection group showed 1689 genes upregulated and 1905 genes downregulated compared to healthy skin. (B) Cellular component in GO analysis of the differentially expressed genes. (C) Biological process in GO analysis of the differentially expressed genes. (D) Molecular function in GO analysis of the differentially expressed genes. (E) Statistics of pathway enrichment in KEGG analysis of the differentially expressed genes.

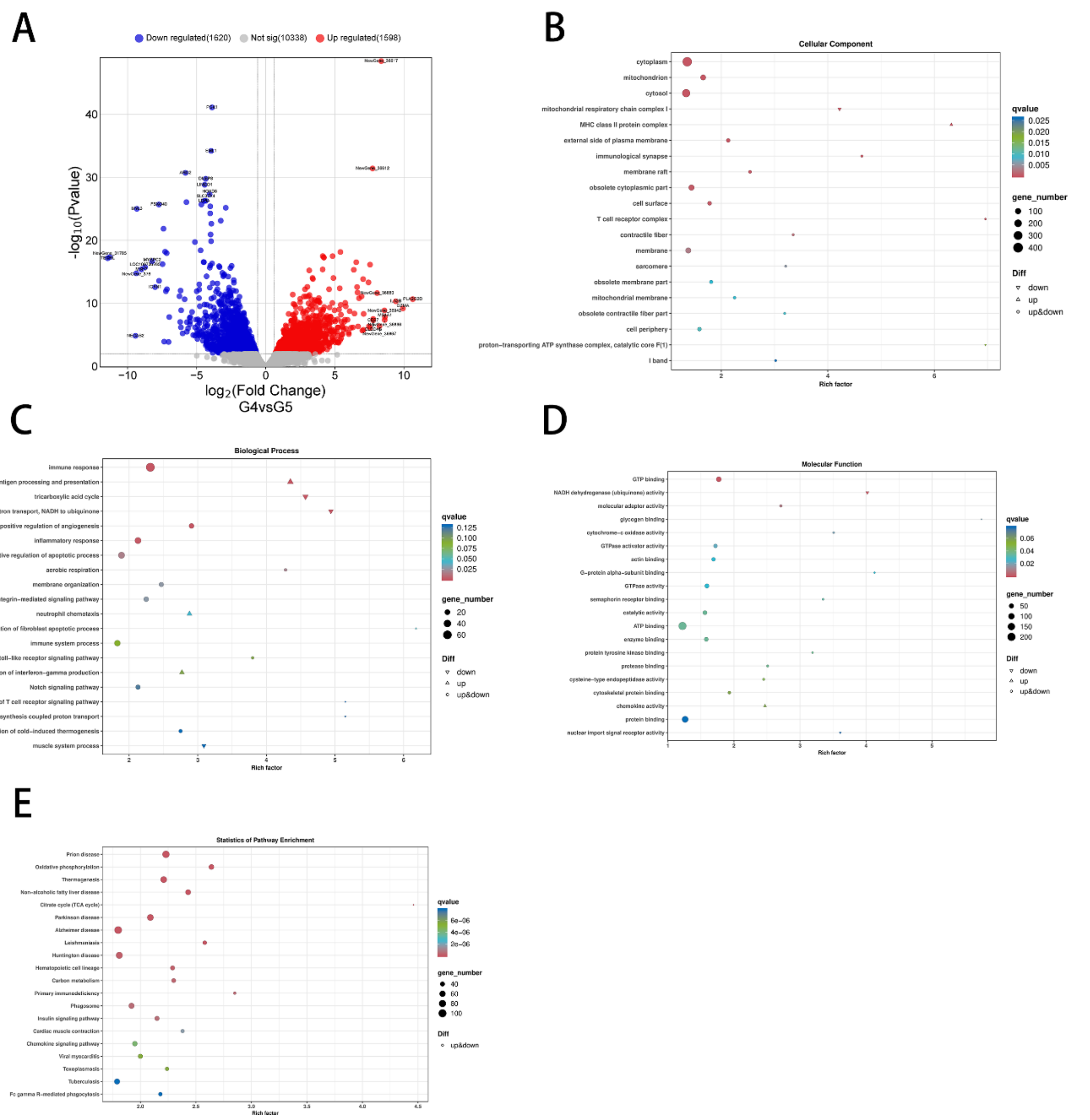

Supplementary Figure 4. Analysis of differentially expressed genes and related pathways between groups 4 and 5. (A) The muscle of the TGMS-TAC injection group exhibited 1598 genes upregulated and 1620 genes downregulated. (B) Cellular component in GO analysis of the differentially expressed genes. (C) Biological process in GO analysis of the differentially expressed genes. (D) Molecular function in GO analysis of the differentially expressed genes. (E) Statistics of pathway enrichment in KEGG analysis of the differentially expressed genes.

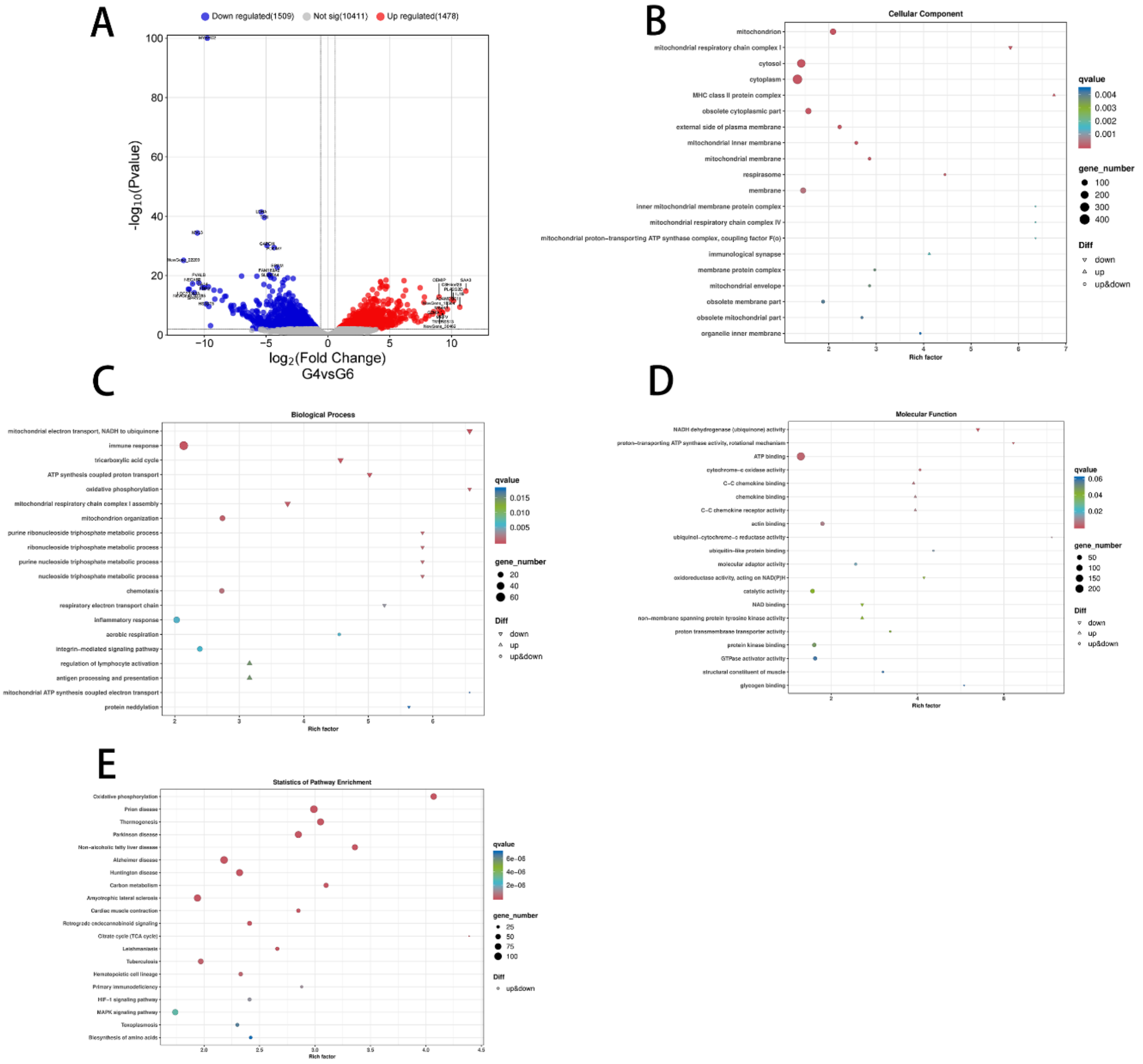

Supplementary Figure 5. Analysis of differentially expressed genes and related pathways between groups 4 and 6. (A) In the muscle tissue of the end-stage rejection group, 1478 genes were upregulated, and 1509 genes were downregulated compared with the healthy muscle group. (B) Cellular component in GO analysis of the differentially expressed genes. (C) Biological process in GO analysis of the differentially expressed genes. (D) Molecular function in GO analysis of the differentially expressed genes. (E) Statistics of pathway enrichment in KEGG analysis of the differentially expressed genes.

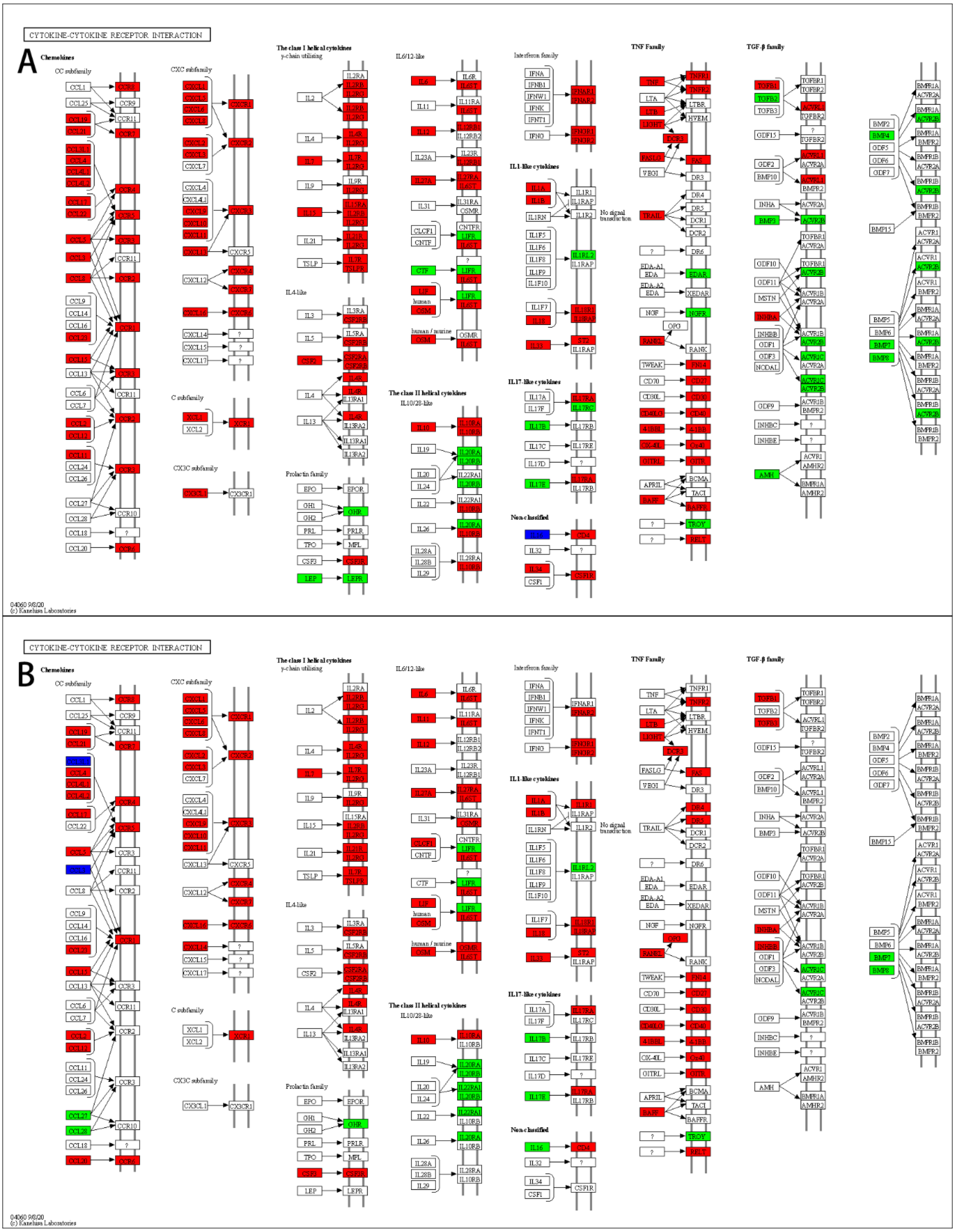

A
CYTOKINE-CYTOKINE RECEPTOR INTERACTION
Chemokines
CC subfamily
CXC subfamily
C subfamily
CX3C subfamily
The class I helical cytokines
γ-chain utilising
IL4-like
Prolactin family
IL6/12-like
The class II helical cytokines
IL10/28-like
Interferon family
IL1-like cytokines
No signal transduction
IL17-like cytokines
Non-classified
TNF Family
TGF-β family
04060 9/8/20
(c) Kanehisa Laboratories
B
CYTOKINE-CYTOKINE RECEPTOR INTERACTION
Chemokines
CC subfamily
CXC subfamily
C subfamily
CX3C subfamily
The class I helical cytokines
γ-chain utilising
IL4-like
Prolactin family
IL6/12-like
The class II helical cytokines
IL10/28-like
Interferon family
IL1-like cytokines
No signal transduction
IL17-like cytokines
Non-classified
TNF Family
TGF-β family
04060 9/8/20
(c) Kanehisa Laboratories

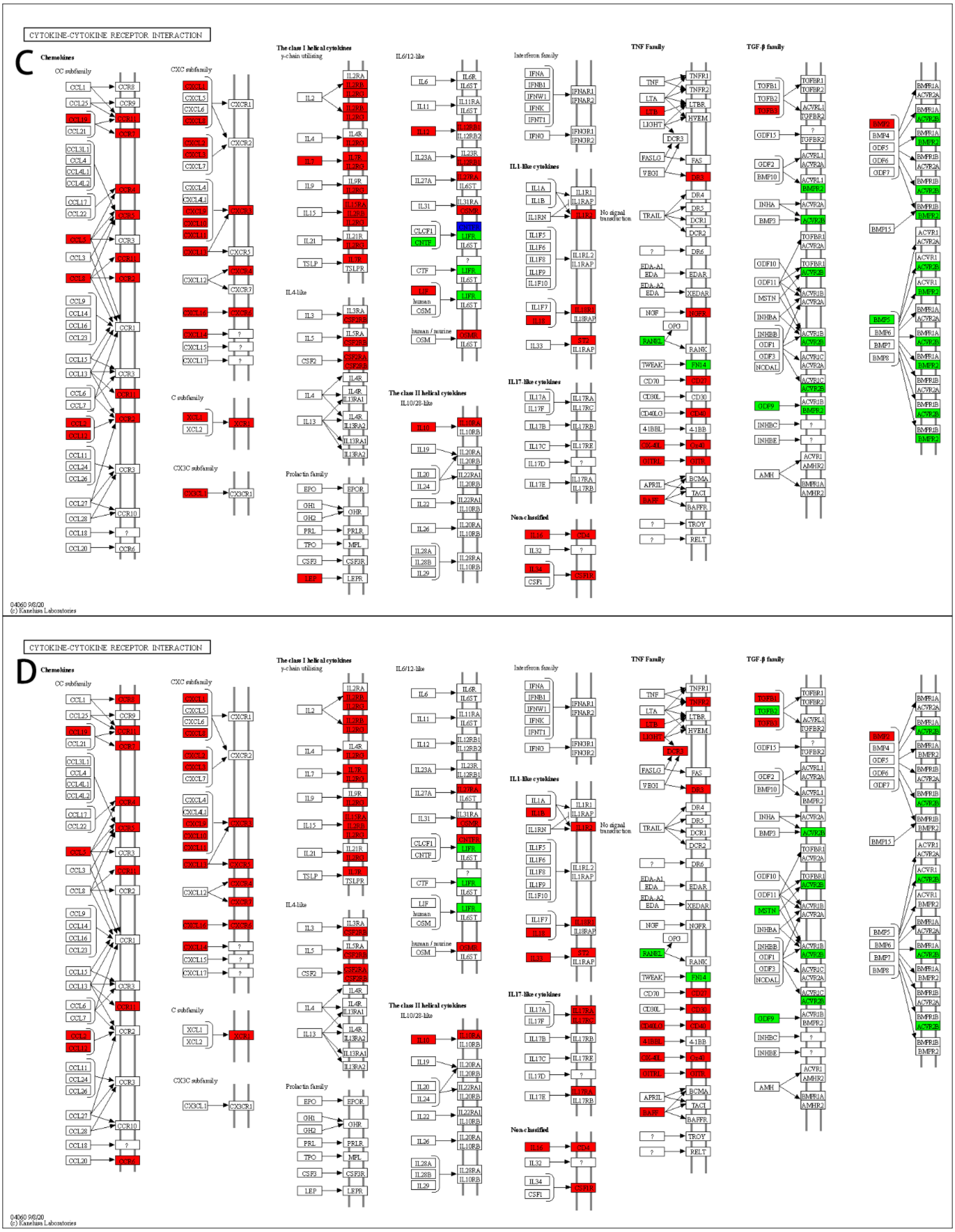

Supplementary Figure 6. The KEGG pathway (mmu04060) for cytokine-cytokine receptor interaction in different group comparisons. (A) Group 1 healthy skin vs group 2 TGMS-TAC injection skin, (B) Group 1 healthy skin vs group 3 end-stage rejection skin, (C) Group 4 healthy muscle vs group 5 TGMS-TAC injection muscle, and (D) Group 4 healthy muscle vs group 6 end-stage rejection muscle. Red denotes up-regulated genes, green represents down-regulated genes, and blue indicates the existence of both up-regulated and down-regulated related genes.

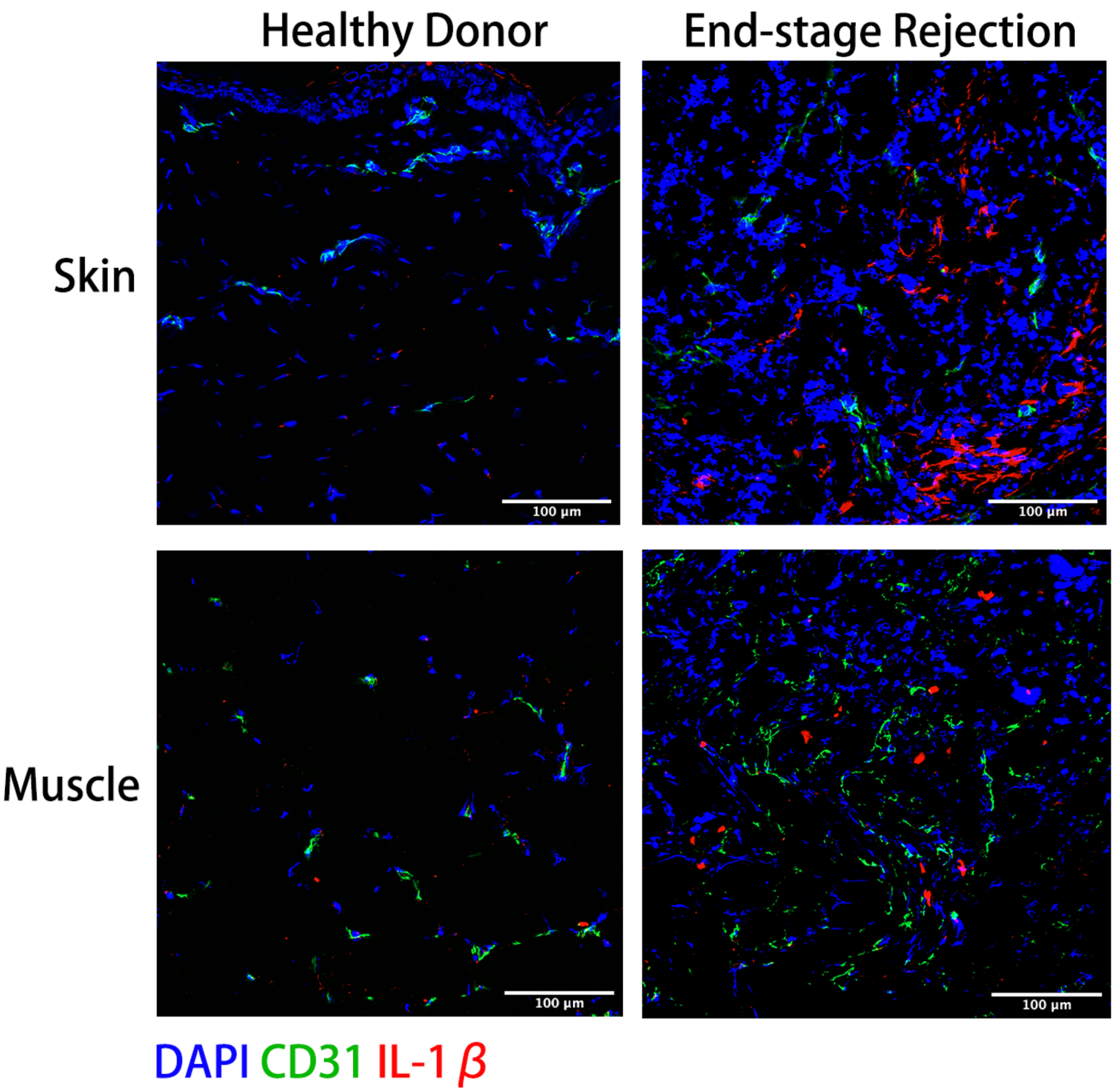

Supplementary Figure 7. Immunofluorescence visualization of IL-1β. The upregulation of IL-1β in skin and muscle of the end-stage rejection groups was shown compared to healthy tissues.

Input Sample	B cells naive	B cells memory	Plasma cells	T cells CD8	T cells CD4 naive	T cells CD4 memory resting	T cells CD4 memory activated	T cells follicular helper	T cells regulatory (Tregs)	T cells gamma delta	NK cells resting	NK cells activated
G1a	0	0.026	0	0.062	0	0.006	0	0.062	0.041	0	0.072	0.049
G1b	0	0.117	0	0.065	0	0	0	0.051	0.03	0	0.134	0
G1c	0.011	0.051	0	0.13	0	0.077	0	0.066	0.027	0	0	0.108
G2a	0.007	0	0.047	0.188	0	0.097	0.015	0.053	0.055	0	0.045	0
G2b	0	0.012	0	0.224	0	0.076	0.005	0.047	0.056	0.009	0.024	0
G2c	0.021	0	0	0.22	0	0.041	0	0.05	0.086	0	0.096	0
G3a	0.017	0	0.01	0.191	0	0.192	0.002	0.095	0.077	0	0.07	0
G3b	0	0.021	0.082	0.131	0	0	0	0.055	0.08	0	0	0.037
G3c	0	0.005	0.017	0.106	0	0.143	0	0.047	0.057	0	0.04	0
G4a	0.053	0	0.062	0.197	0	0.012	0	0.04	0.025	0	0	0.096
G4b	0.001	0	0.033	0.332	0	0.099	0	0	0.003	0	0.054	0.053
G4c	0.083	0	0.039	0.179	0	0.113	0	0.01	0.038	0	0.071	0.076
G5a	0	0.014	0.027	0.178	0	0.096	0.015	0.07	0.095	0.053	0.036	0
G5b	0.044	0	0.075	0.173	0	0.017	0	0.152	0.037	0	0.023	0.039
G5c	0.067	0.064	0.079	0.169	0	0.058	0	0.119	0	0	0.033	0.043
G6a	0.012	0.061	0.041	0.208	0	0.014	0	0.216	0.096	0	0.008	0.049
G6b	0	0.062	0.057	0.158	0	0.054	0	0.059	0.088	0	0.049	0
G6c	0.007	0.009	0.097	0.172	0	0	0	0.08	0.02	0	0.108	0

Input Sample	Monocytes	Macrophages M0	Macrophages M1	Macrophages M2	Dendritic cells resting	Dendritic cells activated	Mast cells resting	Mast cells activated	Eosinophils	Neutrophils	P-value	Correlation
G1a	0.206	0.026	0.085	0.176	0.091	0	0.099	0	0	0.000	0.300	0.056
G1b	0.122	0	0.081	0.253	0.045	0	0.102	0	0	0.000	0.470	0.033
G1c	0.084	0	0.061	0.288	0.046	0	0.049	0	0	0.000	0.390	0.045
G2a	0.081	0.008	0.182	0.123	0.049	0	0.029	0	0.023	0.000	0.000	0.551
G2b	0.044	0	0.205	0.213	0.011	0	0	0.064	0.007	0.003	0.000	0.609
G2c	0.023	0	0.193	0.246	0.008	0	0.015	0	0	0.000	0.000	0.512
G3a	0.024	0.081	0.132	0.057	0.047	0	0.005	0	0	0.000	0.000	0.419
G3b	0.047	0	0.092	0.1	0	0	0	0.31	0	0.045	0.010	0.306
G3c	0.054	0.076	0.158	0.022	0.023	0	0	0.151	0	0.100	0.000	0.405
G4a	0.152	0	0.055	0.274	0	0	0	0.004	0.021	0.008	0.810	-0.011
G4b	0.073	0.036	0.059	0.096	0	0	0.144	0	0.017	0.000	0.950	-0.032
G4c	0.071	0	0.127	0.128	0.023	0	0.041	0	0	0.000	0.960	-0.033
G5a	0.027	0.013	0.194	0.145	0.011	0.023	0	0.002	0	0.000	0.000	0.498
G5b	0.059	0	0.259	0.098	0	0	0.018	0	0.006	0.000	0.000	0.519
G5c	0.004	0	0.213	0.082	0.006	0	0.02	0	0.042	0.000	0.020	0.231
G6a	0.02	0.024	0.191	0	0.018	0.026	0.015	0	0	0.000	0.000	0.319
G6b	0.095	0.023	0.146	0.069	0.04	0	0	0.073	0.025	0.001	0.000	0.353
G6c	0.099	0.029	0.215	0.091	0.043	0	0.002	0	0.028	0.000	0.000	0.378

Supplementary Figure 8. Aggregate scores of immune cell composition across all samples. To enhance our comprehension of the immune cell makeup in VCA rejection tissue, cell-type enrichment analysis using the Cibersort tool with gene expression data for 22 immune cell types was conducted. The findings revealed that the skin and muscle of the end-stage rejection groups showed elevated immune scores in comparison to the healthy skin and muscle tissues.

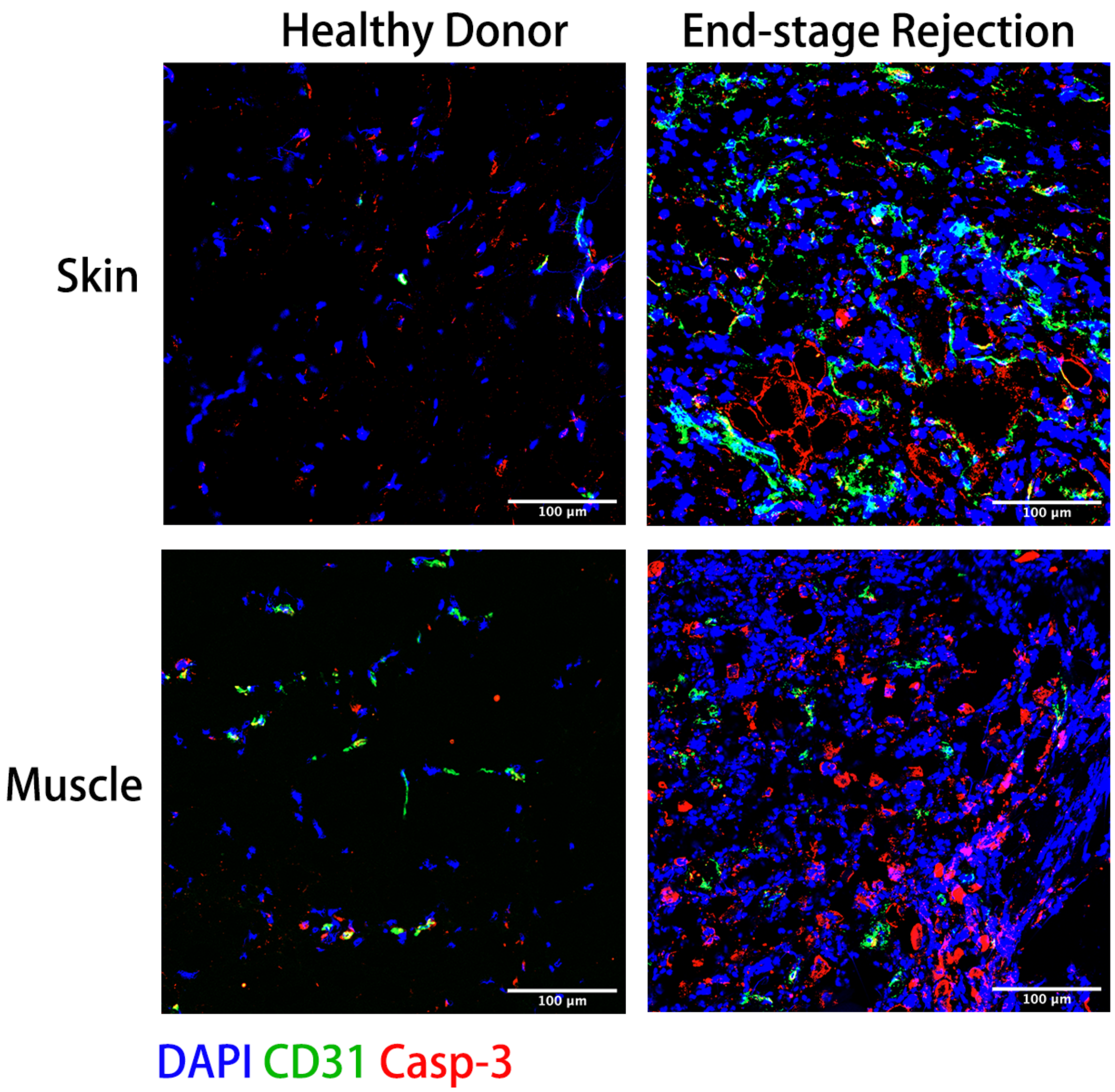

Supplementary Figure 9. Immunofluorescence visualization of caspase-3. The upregulation of caspase-3 in the skin and muscle of the end-stage rejection groups was shown compared to healthy skin and muscle.
